# *Erigeron annuus* (L.) Pers. Extract Inhibits Reactive Oxygen Species (ROS) Production and Fat Accumulation in 3T3-L1 Cells by Activating an AMP-Dependent Kinase Signaling Pathway

**DOI:** 10.3390/antiox8050139

**Published:** 2019-05-20

**Authors:** Yoon-Hee Choi, Ok-Hwan Lee, Yulong Zheng, Il-Jun Kang

**Affiliations:** 1 Department of Food and Nutrition, Hallym Polytechnic University, Chuncheon 24210, Korea; cyh@hsc.ac.kr; 2 Department of Food Science and Biotechnology, Kangwon National University, Chuncheon 24341, Korea; loh99@kangwon.ac.kr; 3 Department of Food Science and Nutrition, Hallym University, Chuncheon 24252, Korea; zyl1994@naver.com

**Keywords:** *Erigeron annuus* (L.) Pers, 3T3-L1 adipocyte, ROS production, fat accumulation, AMPK signaling pathway

## Abstract

Obesity is one of the major public health problems in the world because it is implicated in metabolic syndromes, such as type 2 diabetes, hypertension, and cardiovascular diseases. The objective of this study was to investigate whether *Erigeron annuus* (L.) Pers. (EAP) extract suppresses reactive oxygen species (ROS) production and fat accumulation in 3T3-L1 cells by activating an AMP-dependent kinase (AMPK) signaling pathway. Our results showed that EAP water extract significantly inhibits ROS production, adipogenesis, and lipogenesis during differentiation of 3T3-L1 preadipocytes. In addition, EAP decreased mRNA and protein levels of proliferator-activated receptor γ (PPARγ) and CCAAT/enhancer-binding protein alpha (C/EBPα). Moreover, EAP suppressed mRNA expressions of fatty acid synthase (FAS), lipoprotein lipase (LPL), adipocyte protein 2 (aP2) in a dose-dependent manner. Whereas, EAP upregulated adiponectin expression, phosphorylation levels of AMPK and carnitine palmitoyltransferase 1 (CPT-1) protein level during differentiation of 3T3-L1 preadipocytes. These results suggest that EAP water extract can exert ROS-linked anti-obesity effect through the mechanism that might involve inhibition of ROS production, adipogenesis and lipogenesis via an activating AMPK signaling pathway.

## 1. Introduction

Obesity is a major public health problem around the globe because it is implicated in metabolic syndromes, including type 2 diabetes, and cardiovascular diseases. In 2005, the World Health Organization (WHO) reported that 1.6 billion adults are overweight and 0.4 billion are obese among adults worldwide [1]. High-calories diet and deskbound lifestyle are the most dominant factors contributing to obesity [2]. Due to the rapid increase of obesity-related diseases, cellular and molecular mechanisms underlying fat metabolism need to be clarified. Obesity is affected by both the number and size of adipose tissue that is accelerated by adipogenesis and lipogenesis progression [3,4]. Regulating adipogenesis, therefore, provides a promising therapeutic approach for preventing obesity. Moreover, recent reports have explored the mechanism study of adipocyte life cycles. such as adipogenesis, lipogenesis, and lipolysis using 3T3-L1 cells [5,6].

Expression of cell-specific adipogenic transcription factors in addition to regulation of cell phase together with proliferator-activated receptor γ (PPARγ) and CCAAT/enhancer-binding protein alpha (C/EBPα) can facilitate the expression of genes. Adipocytes differentiation is a convoluted procedure exaggerated through numerous hormones and transcript aspects. Adipogenesis is accurately and forcefully controlled by transcriptional flow where transcript aspects can trigger or suppress each other’s expression in a consecutive method. Transcriptional cascade strategic actors include PPARγ, a nuclear receptor, and C/EBP family members, such as C/EBPα, C/EBPβ, and C/EBPδ, known to be dominant in adipocyte differentiation by both in vitro and in vivo studies [7]. Adipocyte differentiation, C/EBPα, PPARγ, and sterol regulatory element binding protein-1c (SREBP-1c), are reflected strategic controllers in adipogenesis, comprising adiponectin, leptin, lipoprotein lipase (LPL), and adipocyte-specific fatty acid binding protein (FABP) [8,9,10,11]. SREBP-1c expression is known to be controlled by insulin and AMP-dependent kinase (AMPK) signaling pathway [12,13]. AMPK is one of the most important targets to inhibit and control obesity [14,15]. AMPK can control cellular metabolism and glucose and lipid metabolism, thus controlling energy. AMPK can rapidly activate the uptake, storage, and metabolism of numerous substrates after it is activated in adipose tissue. Moreover, AMPK can activate fatty acid β-oxidation by inactivating acetyl-CoA carboxylase (ACC) and upregulating the expression of carnitine palmitoyltransferase 1 (CPT1) [16].

Oxidative stress reasons many responses, as well as degradation of numerous fragments and alteration of intracellular indicator transduction. In the course of the adipogenesis, adipocytes as well as produce variation adipogenic cytokines, ROS and fatty acids [17]. Recent studies suggest that fat cell-derivative ROS roles as a key regulator of metabolic syndrome, comprising insulin conflict and obesity [18,19]. Furthermore, ROS productions plays a serious starring role in the instruction of adipocyte differentiation related to the improved adipogenic transcription factors gene expressions [20]. The current study proposes that the escalation of cellular ROS level is a common sign in the pathological complaint, such as lipid accumulation [18]. Thus, lipid homeostasis is sustained by the fine-turning of lipogenesis and lipolysis, it is controlled by accommodating feat of numerous enzymes in the metabolic organs, principally adipose tissues, the liver, and muscles. 

*Erigeron annuus* (L.) Pers. (EAP) is a biennial plant belonging to the daisy family and is native to North America. It also grows widely in Korea near mountains and lakes as a naturalized plant. It has been used to treat multiple disorders, e.g., cyanotic detoxification, acute gastroenteritis, acute infectious hepatitis, schizophrenia, hypoglycemia, and anti-inflammation [21,22]. Recent studies have revealed that EAP possesses functions of antimicrobial, antitumor, anti-inflammatory, antioxidant, and cellular imperviousness-improving effects [23,24]. However, the anti-obesity effect of EAP has not been studied yet. Thus, the objective of this study was to elucidate the effect of EAP on ROS production and lipid accumulation in the differentiation of 3T3-L1 preadipocytes by measuring levels of adipocyte-specific transcription factors such as C/EBPα, PPARγ, and phosphorylated AMPK and ACC.

## 2. Materials and Methods 

### 2.1. Reagents

Dulbecco’s modified Eagle’s medium (DMEM) was purchased from Lonza (Cologne, Germany). Fetal bovine serum (FBS), bovine calf serum (BCS), insulin, and penicillin-streptomycin (P/S) were purchased from Gibco (Gaithersburg, MD, USA). Nitro blue tetrazolium (NBT), dexamethasone and 3-isobutyl-1-methylxanthine (IBMX) were purchased from Sigma Chemical Co. (St. Louis, MO, USA). All other reagents of the highest grade available were obtained from commercial sources. WST-1 was purchased from Roche Molecular Biochemicals (Mannheim, Germany). 

### 2.2. Preparation EAP Extracts

*Erigeron annuus* (L.) Pers. (EAP) sample was collected locally in Chuncheon, Kangwon-Do, Korea. Leaves and stems of EAP were subjected to natural drying at room temperature. These dried leaves and stems of EAP (488.73 g) were crushed to pieces. Crushed leaves and stems were extracted three times with various extraction solvents (water, 30%-ethanol, 50%-ethanol, 70%-ethanol, and 100%-ethanol) for 3 h. The extract was concentrated with a vacuum evaporator (Rotavapor R-220, Buchi, Flawil, Switzerland), followed by freeze-drying with a freeze-drier (Il Shin Lab Co., Seoul, Korea) to obtain powder (water extract: 21.8 g, 30%-ethanol extract: 20.4 g, 50%-ethanol extract: 25.7 g, 70%-ethanol extract: 23.3 g, 100%-ethanol extract: 10.2 g). Extraction yields of water extract, 30%-ethanol extract, 50%-ethanol extract, 70%-ethanol extract, and 100%-ethanol extract of EAP were 27.3%, 25.6%, 32.2%, 29.2%, and 12.8%, respectively.

### 2.3. Cell Culture and Differentiation

3T3-L1 cells were purchased from the ATCC. Cells were seeded into 24-well plates and were cultured in DMEM containing 10% BCS and 1% P/S at 37 °C with 5% CO_2_. Two days after complete confluence was reached (D0), cells were cultured in MDI differentiation medium (DMEM containing 0.5 mM/L IBMX, 1 μM/L dexamethasone, and 10 μg/mL insulin), 1% PS, and 10% FBS for three days (D3). To evaluate the effects of EAP extract on adipocyte differentiation of 3T3-L1 preadipocytes, cells were cultured in MDI in the presence of various concentrations of EAP extract. Cells were then maintained in regular medium containing 1% PS, 10% FBS, and 10 μg/mL insulin (D5). After five days of induction, the medium was changed to DMEM containing 1% PS and 10% FBS (D7). On day seven, cells were harvested for further experiment.

### 2.4. Cell Viability Assay

Cell viability of 3T3-L1 preadipocytes and adipocytes was assessed using WST-1 assay. 3T3-L1 preadipocyte and adipocytes cells were incubated with EAP extracts (0–300 μg/mL) for 24 h and seven days, respectively. WST-1 was added to the cultured medium, followed by incubation for 2 h. Cell viability was measured in absorbance at a wavelength of 570 nm.

### 2.5. NBT Assay and Oil Red O (ORO) Staining

The effect of the EAP on ROS production was determined by NBT assay during the differentiation of 3T3-L1 cells. NBT is reduced by ROS to a dark-blue, insoluble form of NBT called formazan. On day seven after induction, the cells were incubated for 90 min in PBS containing 0.2% NBT. Formazan was dissolved in 50% acetic acid, and the absorbance was determined at 570 nm.

The effect of the EAP on fat accumulation in 3T3-L1 cells was evaluated by ORO staining. 3T3-L1 cells were fixed with 4% formaldehyde in PBS for 1 h at room temperature and washed twice with 60% isopropanol. After performing ORO staining for 1 h at room temperature, cells were washed with water to remove the excess stain. Stained cells were allowed to air dry and ORO stained cells were eluted with DMSO for quantitative analysis. The absorbance was measured at a wavelength of 490 nm on a spectrophotometer.

### 2.6. Real-Time Polymerase Chain Reaction (RT-PCR)

Total RNA was isolated from cells after seven days of maturation using high pure RNA isolation kit (Roche Applied Science, Penzberg, Germany). NanoDrop (NanoDrop 2000c, Thermo Scientific, Waltham, MA, USA) was used to quantify total RNA. Then 1 μg of total RNA was converted into cDNA using a cDNA synthesis kit (Roche Applied Science, Penzberg, Germany). Real-time quantitation was performed using Light Cycler 480 (Roche Diagnostics, Manneim, Germany). PCR reaction mix contained Light Cycler 480 SYBR Green I Master (Roche, Germany). The real-time PCR conditions were as follows: 95 °C for 10 min followed by forty-five cycles at 95 °C for 15 s, 60 °C for 5 s, 72 °C for 15 s. All experiments were performed three or more times. Expression levels of target genes were normalized against glyceraldehyde-3-phosphate dehydrogenase (GAPDH) or β-actin as internal controls. Primers used in the experiment are shown in Table 1.

### 2.7. Analysis of Protein Level

Cells were harvested using a cell scraper and lysed to obtain whole cell lysate. Cells lysates were clarified by centrifugation at 12,000 g for 30 min. Protein concentrations were measured with bicinchoninic acid (BCA) protein assay kit (Pierce Biotechnology, Waltham, MA, USA). Then 20 μg of the protein extract was mixed with 2 × sample buffer, heated at 95 °C for 5 min, separated by 10% SDS-PAGE, and transferred to PVDF membrane at 100 V for 90 min. Membranes were blocked with 1 × TBST comprising 5% skim milk at room temperature for 1 h, incubated with primary antibody overnight at 4 °C, washed five times with 1 × TBST (10 min each wash), incubated with secondary antibody at room temperature for 1 h, and washed five times with 1 × TBST (10 min each wash). PPARγ, C/EBPα, SREBP-1c, phospho-AMPK (*p*-AMPK), total-AMPK, and phospho-ACC (*p*-ACC), CPT1 antibodies were purchased from Cell Signaling Technology (Beverly, MA, USA). The target protein was detected with Luminata ™ Forte Western HRP substrate (Millipore, Tokyo, Japan). The density of a specific band was analyzed using image J software (NIH, Bethesda, MD).

### 2.8. Statistical Analysis

Experimental results are presented as mean ± standard deviation (SD) of three experiments. All results were statistically analyzed by Duncan’s multivariate analysis. Difference between averages was considered statistically significant when the *p*-value was less than 0.05.

## 3. Results

### 3.1. EAP Water Extract Inhibits Lipid Accumulation and Ros Production in 3t3-l1 Adipocytes

**Figure 1:**
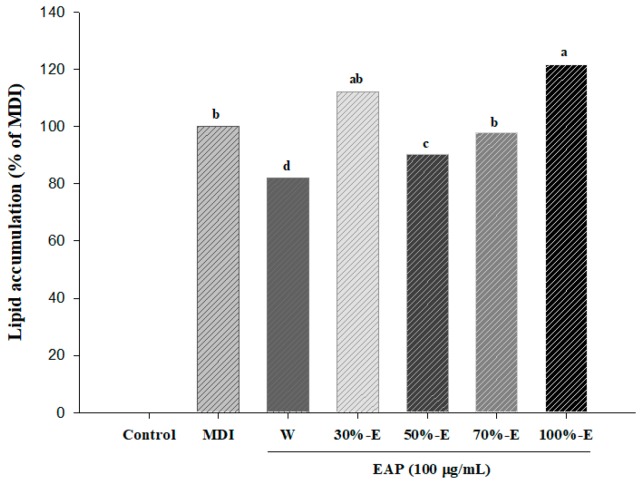
Effects of various extracts of EAP on lipid accumulation in 3T3-L1 adipocytes. EAP: *Erigeron annuus* (L.) Pers. water extract, Post-confluent 3T3-L1 preadipocytes were differentiated in the presence of various EAP extracts (water, 30% ethanol, 50% ethanol, 70% ethanol and 100% ethanol) for seven days. Lipid accumulation of cells was determined by Oil Red O (ORO) staining followed by reading absorbance at 490 nm. Values are presented as mean ± standard deviation. Differences between means were considered statistically significant at *p* < 0.05.

To observe effects of EAP extracts on adipocytes differentiation, 3T3-L1 cells were treated with MDI in the presence or absence of various EAP extracts (water, 30%-ethanol, 50%-ethanol, 70%-ethanol, and 100%-ethanol) at a concentration of 100 μg/mL. Among the various EAP extracts, water extract has the greatest inhibitory effect on adipogenesis (Figure 1). Thus, it was further analyzed for its effect on adipocyte differentiation and molecular mechanisms involved in its effect. 

**Figure 2:**
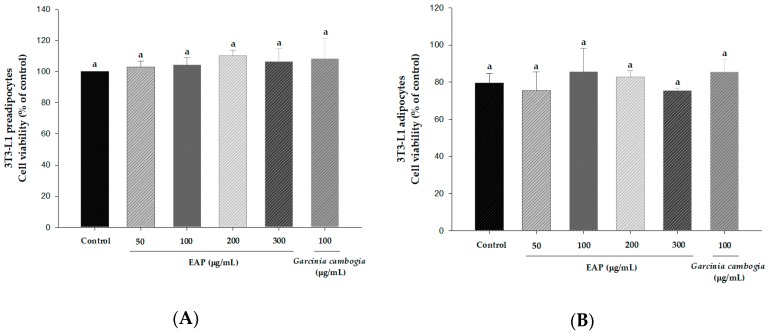
Effects of EAP water extract on cell viability of 3T3-L1 preadipocytes (**A**) and adipocytes (**B**). 3T3-L1 cells were treated with EAP water extract at various concentrations (50, 100, 200, and 300 μg/mL) for 24 h (**A**) and seven days (**B**). Cell viability was measured by WST-1 assay. Values are presented as mean ± standard deviation. Differences between means were considered statistically significant at *p* < 0.05.

**Figure 3:**
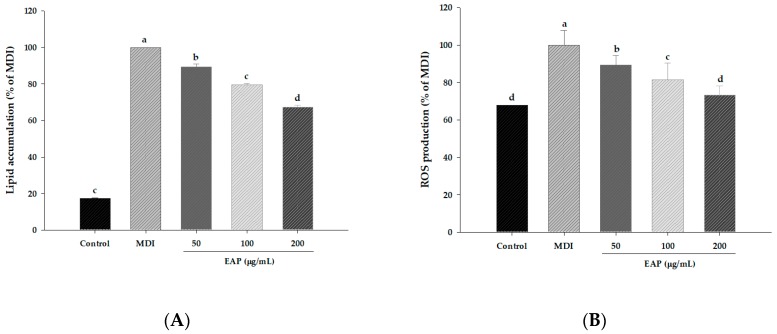
Effects of EAP water extract on lipid accumulation (**A**) and ROS production (**B**) in 3T3-L1 adipocytes. EAP: *Erigeron annuus* (L.) Pers. water extract, 3T3-L1 preadipocytes were induced to differentiate in the presence of EAP (added on D0 of differentiation) for seven days. (**A**) Lipid accumulation was determined by measuring absorbance at 490 nm. (**B**) ROS production was determined by using nitro tetrazolium blue chloride and absorbance at 570 nm. Values are presented as mean ± standard deviation. Differences between means were considered statistically significant at *p* < 0.05.

To evaluate the effect of EAP water extract on the cell viability of preadipocyte and adipocytes, cultured 3T3-L1 cells were treated with various concentrations of EAP water extract and cultured for 24 h or seven days followed by cell viability using WST-1. Treatment with EAP water extract did not significantly affect cell viability (Figure 2). Thus, we used this extract at up to 200 μg/mL in subsequent experiments. We further determined the effect of EAP water extract on lipid accumulation and ROS production in 3T3-L1 adipocytes by ORO staining and NBT assay. EAP water extract treatment dose-dependently inhibits lipid accumulation during adipogenesis (Figure 3). Moreover, the production of dark-blue formazan, which represents ROS production, was decreased in adipocytes treated with EAP water extract when compared with MDI treated cells (Figure 3).

### 3.2. Effect of EAP Water Extract on Adipogenic and Lipogenic Gene Expressions

**Figure 4:**
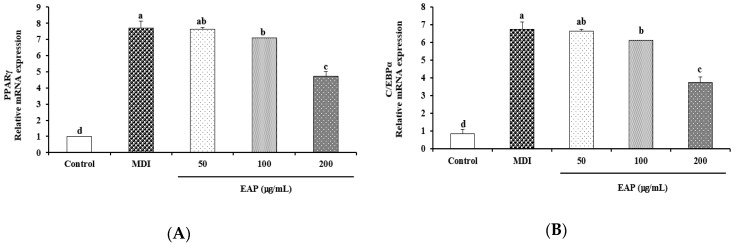
Effects of EAP on mRNA expression of PPARγ (**A**) and C/EBPα (**B**) in 3T3-L1 adipocytes. EAP: *Erigeron annuus* (L.) Pers. water extract, 3T3-L1 preadipocytes were differentiated in the presence of EAP at different concentrations (50 to 200 μg/mL) for seven days. The mRNA expression level was measured by real time-PCR and normalized using housekeeping gene GAPDH. Values are presented as mean ± standard deviation. Differences between means were considered statistically significant at *p* < 0.05.

**Figure 5:**
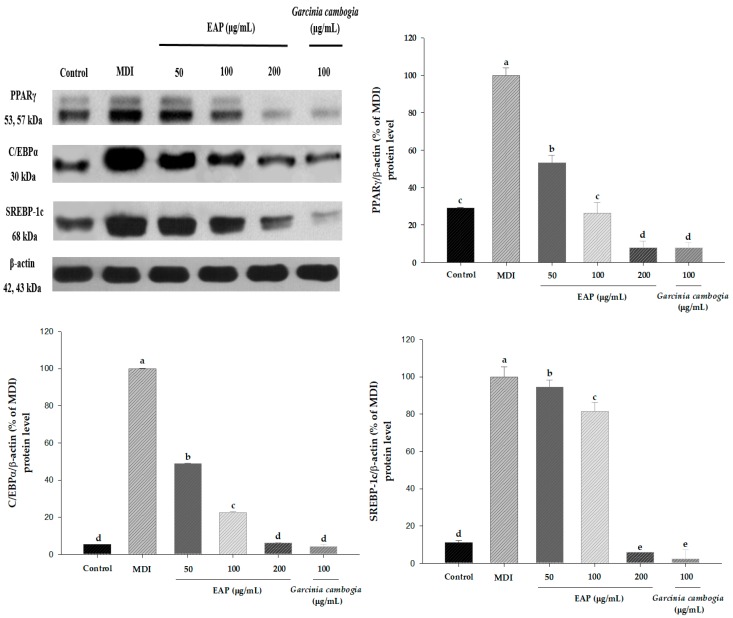
Effects of EAP on protein levels of PPARγ, C/EBPα and SREBP-1c in 3T3-L1 adipocytes. EAP: *Erigeron annuus* (L.) Pers. water extract. These cells were treated with EAP (from 50 to 200 μg/mL) for seven days during differentiation. (a) Western blot analysis of PPARγ, C/EBPα, and SREBP-1c. At day seven, total protein was harvested. (b–d) Protein levels were determined by western blotting. Values are presented as mean ± standard deviation. Differences between means were considered statistically significant at *p* < 0.05.

We evaluated the effect of EAP water extract on mRNA and protein expression of adipogenic target gene during adipogenesis of 3T3-L1 cells using real-time RT-PCR and western blot. Our results revealed that EAP water extract at a concentration of 200 μg/mL significantly downregulated the mRNA expression of PPARγ and C/EBPα (Figure 4). Moreover, EAP dose-dependently suppressed protein levels of PPARγ, C/EBPα and SREBP-1c in 3T3-L1 cell parallel with mRNA expression of PPARγ and C/EBPα gene. *Garcinia cambogia* at a concentration of 100 μg/mL, used as the positive control, decreased protein levels of PPARγ, C/EBPα and SREBP-1c in 3T3-L1 cell (Figure 5). These results indicate that EAP water extract exerts the anti-adipogenic effect by blocking PPARγ and CEBPα expression, which might have implications in anti-obesity effects.

**Figure 6:**
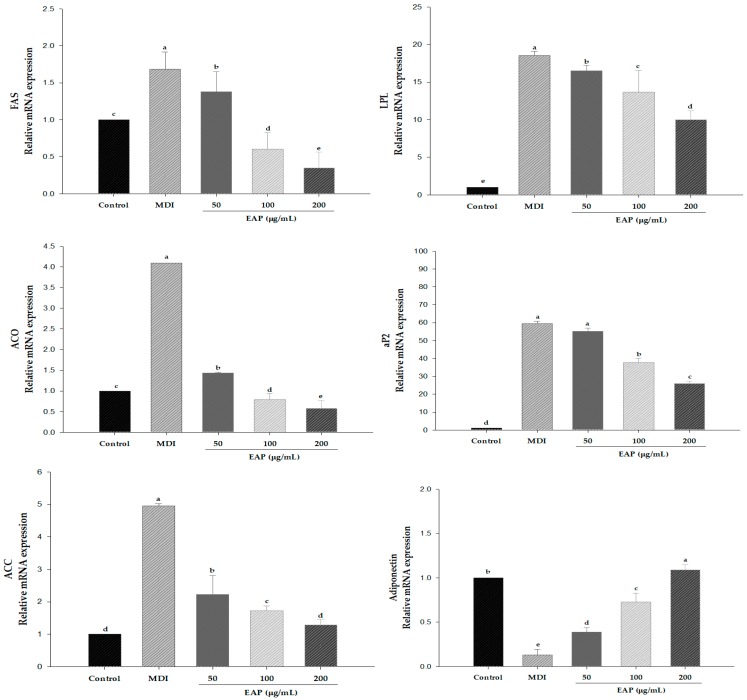
Effects of EAP on mRNA expressions of FAS, LPL, aP2, ACO, ACC and adiponectin genes in 3T3-L1 adipocytes. EAP: *Erigeron annuus* (L.) Pers. water extract. Cells were treated with EAP (50, 100, and 200 μg/mL) for seven days during differentiation. At day seven, mRNA expression levels of FAS, LPL, aP2 and adiponectin were determined by real-time PCR. Results are expressed as fold increase compared to the control after normalizing against GAPDH expression level. Values are presented as mean ± standard deviation. Differences between means were considered statistically significant at *p* < 0.05.

In order to confirm whether this inhibitory effect of EAP water extract on lipid accumulation is mediated through inhibition of adipogenesis and lipogenesis involving PPARγ, C/EBPα, and SREBP-1c, we further checked the FAS, LPL, aP2 and adiponectin gene expressions using RT-PCR. As shown in Figure 6, EAP water extract markedly suppressed mRNA expression of fatty acid synthase (FAS), LPL, and adipocyte protein 2 (aP2) in a dose-dependent manner. However, EAP water extract dose-dependently increased adiponectin gene expression. These results indicated that EAP water extract inhibits lipid accumulation and ROS production in 3T3-L1 adipocytes via inhibition of adipogenesis and lipogenesis. 

### 3.3. EAP Water Extract Enhances AMPK Phosphorylation and Its Downregulation ACC

**Figure 7:**
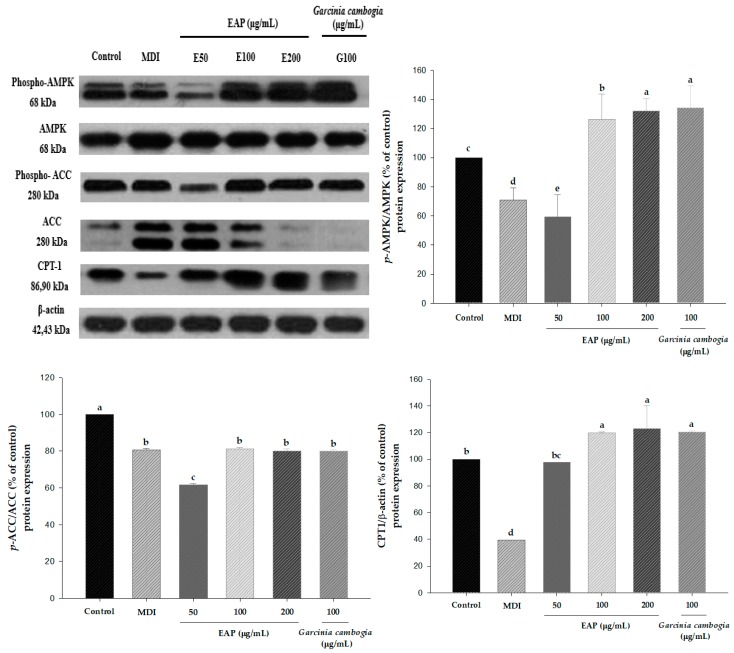
Effects of EAP on the activation of AMPK signaling in 3T3-L1 adipocytes. EAP: *Erigeron annuus* (L.) Pers. water extract. Cells were treated with EAP at 200 μg/mL during MDI-induced adipocytes. *p*-AMPK, AMPK, *p*-ACC, ACC, and CPT1 were determined by Western blot analysis. Band densities of *p*-AMPK and *p*-ACC were quantified and normalized with those of AMPK and ACC expression levels, respectively. Values are presented as mean ± standard deviation. Differences between means were considered statistically significant at *p* < 0.05.

To elucidate how EAP water extract inhibited lipid accumulation and ROS production during the adipocyte differentiation, AMPK phosphorylated protein level and ACC were measured. We determined whether EAP (200 μg/mL) controlled the differentiation of adipocytes and vitality metabolism via the AMPK pathway in 3T3-L1 cells. The results show that EAP enhances AMPK phosphorylation and ACC in MDI-induced adipocyte differentiation. In addition, EAP upregulated CPT1 protein level (Figure 7). *Garcinia cambogia* also upregulated both phosphorylation AMPK and ACC as well as CPT1 protein levels in 3T3-L1 cells. These results indicated that EAP water extract may inhibit adipogenesis and lipogenesis via an activating AMPK signaling pathway.

## 4. Discussion

*Erigeron annuus* (L.) Pers. (EAP) is a naturalized plant belonging to the daisy family. EAP has been used to treat a variety of diseases in Korea, Japan, and China, including bronchitis, cough, and convulsions [17,18]. However, the antiobesity activity of EAP has not been reported yet. This study evaluated the effect of EAP water extract on lipid accumulation and ROS production in 3T3-L1 cells. 3T3-L1 preadipocyte cells were treated with EAP water extract at varying concentrations. To further clarify the mechanisms involved in this effect, we investigated the effects of EAP on expression levels of adipogenic and lipogenic target gene as well as the AMPK signaling pathway. 

Our data demonstrated that EAP water extract attenuated the lipid accumulation by up to 67.4% compared to MDI-treated control cells (Figure 3). Treatment with EAP water extract also dose-dependently inhibits mRNA and protein expression of PPARγ and C/EBPα paralleled to reduced lipid accumulation in adipocytes (Figure 5). During differentiation of preadipocyte, adipogenesis is activated through the action of transcription factors, such as PPARγ, C/EBPα, and SREBP-1c [25]. SREBP-1c is one of the earliest lipogenic genes besides LPL and FAS [26,27]. In the present study, EAP water extract can inhibit adipogenesis and lipogenesis by downregulating adipogenic and lipogenic markers. Recent studies have shown that C/EBPα, C/EBPβ, and C/EBPδ can also regulate adipocyte gene expression. In the early stage of adipocyte differentiation, C/EBPβ and C/EBPδ activation can increase the expression of C/EBPα, PPARγ, and other lipogenic agents [28,29]. We found that mRNA expression levels of FAS, LPL, and aP2 in 3T3-L1 cells after EAP treatment were significantly downregulated compared to those in DW-treated cells (Figure 6). LPL is linked to ACC and the hydrolysis of plasma triglycerides that contributes to fatty acid synthesis [30]. FAS is a lipogenic enzyme which is an important enzyme catalyzing the last step of fatty acid synthesis [31]. aP2 can stimulate adipogenesis which is controlled by C/EBPα and PPARγ transcriptional levels [32]. aP2 gene is vital to pathways linking obesity to insulin resistance. These results indicate that EAP water extract can prevent MDI-induced expression of a gene linked to adipogenesis and lipogenesis. 

The major regulator of energy metabolism is AMPK. Phosphorylated AMPK plays a role in the regulation of adipocyte differentiation [33]. AMPK has evolved to become a motivational target, especially for the treatment of obesity. It is possible to regenerate a key to inhibit lipogenesis of 3T3-L1 cells by natural complexes [34]. For this reason, AMPK modulation has been predicted to be a key to controlling obesity based on scientific research. We elucidated that EAP water extract could inhibit adipogenesis and phosphorylation level of AMPK and substrate ACC. AMPK can lead to fatty acid β-oxidation through inactivation of ACC and aggregate CPT1 expression gene in adipose tissue [16]. We found that EAP (200 μg/mL) could control the differentiation of adipocytes and vitality metabolism via the AMPK pathway in 3T3-L1 cells. EAP could enhance AMPK phosphorylation and ACC through MDI-induced adipocytes differentiation. EAP also upregulated CPT1 gene expression (Figure 7). These results suggest that EAP water extract inhibits lipid accumulation via activating AMPK signaling pathway. 

In conclusion, our data demonstrate that EAP water extract inhibits lipid accumulation and ROS production in 3T3-L1 cells. Moreover, EAP water extract is capable of inhibiting adipocytes differentiation via downregulation of expression levels of C/EBPα and PPARγ. Furthermore, EAP water extract promotes AMPK phosphorylation and its downstream ACC. Phosphorylated AMPK also increases the expression of the fatty acid oxidase CPT1 gene. Based on these findings, EAP water extract may be useful as an effective natural product to prevent obesity and obesity-related metabolic syndrome. Future identifying studies on bioactive compounds in EAP water extract and in vivo tests using animal HFD-induced obesity models are needed to examine whether EAP water extract could be used as a therapeutic agent and developed as an anti-obesity material, such as a functional food.

## Figures and Tables

**Table 1:** Description of target genes and their primer sequences used for real-time polymerase chain reaction (PCR).

Gene Name	Accession No.	Forward Primer	Reverse Primer
PPARγ ^1^	NM_011146	CGCTGATGCACTGCCTATGA	AGAGGTCCACAGAGCTGATTCC
C/EBPα ^2^	BC058161	CGCAAGAGCCGAGATAAAGC	CACGGCTCAGCTGTTCCA
aP2 ^3^	NM_024406	CATGGCCAAGCCCAACAT	CGCCCAGTTTGAAGGAAATC
ACC ^4^	NM_133360	GAATCTCCTGGTGACAATGCTTATT	GGTCTTGCTGAGTTGGGTTAGCT
FAS ^5^	NM_007988	CTGAGATCCCAGCACTTCTTGA	GCCTCCGAAGCCAAATGAG
LPL ^6^	NM_008509	ATCGGAGAACTGCTCATGATGA	CGGATCCTCTCGATGACGAA
Adiponectin	NM_009605	ATCCACACGTGTACTCAC	AGCATGGTCTACTTCCAG
SPEBP-1c ^7^	NM_011480.3	GGACGAGCTGGCCTTCGGTGA	ATAGGGGGCGTCAAACAGGCCC
GAPDH ^8^	BC083080	GTATGACTCCACTCACGGCAAA	GGTCTCGCTCCTGGAAGATG

^1^ PPARγ, peroxisome proliferator-activated receptor γ. ^2^ C/EBPα, CCAAT/enhancer binding protein α. ^3^ aP2, adipocyte protein 2. ^4^ ACC, acetyl-CoA carboxylase. ^5^ FAS, fatty acid synthase. ^6^ LPL, lipoprotein lipase. ^7^ SREBP-1c, sterol regulatory element binding protein-1c. ^8^ GADPH, glyceraldehydes-3-phosphate dehydrogenase.

## References

[1] Kahn B.B., Flier J.S. (2000). Obesity and insulin resistance. J. Clin. Investig..

[2] Kopelman P.G. (2000). Obesity as a medical problem. Nature.

[3] Harmon A.W., Harp J.B. (2001). Differential effects of flavonoids on 3T3-L1 adipogenesis and lipolysis. Am. J. Physiol. Cell Physiol..

[4] Hirosumi J., Tuncman G., Chang L., Gorgun C.Z., Uysal K.T., Maeda K., Karin M., Hotamisligil G.S. (2002). A central role for JNK in obesity and insulin resistance. Nature.

[5] Mokdad A.H., Ford E.S., Bowman B.A., Dietz W.H., Vinicor F., Bales V.S., Marks J.S. (2003). Prevalence of obesity, diabetes, and obesity-related health risk factors, 2001. JAMA.

[6] Jeon W.J., Oh J.S., Park M.S., Ji G.E. (2013). Anti-hyperglycemic effect of fermented ginseng in type 2 diabetes mellitus mouse model. Phytother. Res..

[7] Park H.J., Della-Fera M.A., Hausman D.B., Rayalam S., Ambati S., Baile C.A. (2009). Genistein inhibits differentiation of primary human adipocytes. J. Nutr. Biochem..

[8] Xu H., Barnes G.T., Yang Q., Tan G., Yang D., Chou C.J., Sole J., Nichols A., Ross J.S., Tartaglia L.A. (2003). Chronic inflammation in fat plays a crucial role in the development of obesity-related insulin resistance. J. Clin. Investig..

[9] Lin J., Della-Fera M.A., Baile C.A. (2005). Green tea polyphenol epigallocatechin gallate inhibits adipogenesis and induces apoptosis in 3T3-L1 adipocytes. Obes. Res..

[10] Rayalam S., Della-Fera M.A., Baile C.A. (2008). Phytochemicals and regulation of the adipocyte life cycle. J. Nutr. Biochem..

[11] Park S.Y., Hwang J.T., Lee Y.K., Kim Y.M., Park O.J. (2009). AMP-activated Kinase Regulates Adipocyte Differentiation Process in 3T3-L1 Adipocytes Treated with Selenium. J. Life Sci..

[12] Sowers J.R. (1998). Obesity and cardiovascular disease. Clin. Chem..

[13] Shojima N., Sakoda H., Ogihara T., Fujishiro M., Katagiri H., Anai M., Onishi Y., Ono H., Inukai K., Abe M. (2002). Humoral regulation of resistin expression in 3T3-L1 and mouse adipose cells. Diabetes.

[14] Lee S.J., Umano K., Shibamoto T., Lee K.G. (2005). Identification of volatile components in basil (Ocimum basilicum L.) and thyme leaves (Thymus vulgaris L.) and their antioxidant properties. Food Chem..

[15] Umar A., Imam G., Yimin W., Kerim P., Tohti I., Berke B., Moore N. (2010). Antihypertensive effects of Ocimum basilicum L. (OBL) on blood pressure in renovascular hypertensive rats. Hypertens Res..

[16] Steppan C.M., Bailey S.T., Bhat S., Brown E.J., Banerjee R.R., Wright C.M., Patel H.R., Ahima R.S., Lazar M.A. (2001). The hormone resistin links obesity to diabetes. Nature.

[17] Turnbaugh P.J., Ley R.E., Mahowald M.A., Magrini V., Mardis E.R., Gordon J.I. (2006). An obesity-associated gut microbiome with increased capacity for energy harvest. Nature.

[18] Iizuka K., Miller B., Uyeda K. (2006). Deficiency of carbohydrate-activated transcription factor ChREBP prevents obesity and improves plasma glucose control in leptin-deficient (ob/ob) mice. Am. J. Physiol. Endocrinol. Metab..

[19] Scagelb J.L.C.F. (2010). Chicoric acid levels in commercial basil (*Ocimum basilicum*) and Echinacea purpurea products. J. Funct. Foods.

[20] Kim E.J., Jung S.N., Son K.H., Kim S.R., Ha T.Y., Park M.G., Jo I.G., Park J.G., Choe W., Kim S.S. (2007). Antidiabetes and antiobesity effect of cryptotanshinone via activation of AMP-activated protein kinase. Mol. Pharmacol..

[21] Kim H.K., Nelson-Dooley C., Della-Fera M.A., Yang J.Y., Zhang W., Duan J., Hartzell D.L., Hamrick M.W., Baile C.A. (2006). Genistein decreases food intake, body weight, and fat pad weight and causes adipose tissue apoptosis in ovariectomized female mice. J. Nutr..

[22] Kim S.O., Yun S.J., Lee E.H. (2007). The water extract of adlay seed (Coix lachrymajobi var. mayuen) exhibits anti-obesity effects through neuroendocrine modulation. Am. J. Chin. Med..

[23] Kropski J.A., Keckley P.H., Jensen G.L. (2008). School-based obesity prevention programs: An evidence-based review. Obes. (Silver Spring).

[24] Luo Q., Li Y., Deng J., Zhang Z. (2015). PARP-1 inhibitor sensitizes arsenic trioxide in hepatocellular carcinoma cells via abrogation of G2/M checkpoint and suppression of DNA damage repair. Chem. Biol. Interact..

[25] Tsai P.J., Davis J., Bryant-Greenwood G. (2015). Systemic and placental leptin and its receptors in pregnancies associated with obesity. Reprod. Sci..

[26] Weisberg S.P., McCann D., Desai M., Rosenbaum M., Leibel R.L., Ferrante A.W. (2003). Obesity is associated with macrophage accumulation in adipose tissue. J. Clin. Investig..

[27] Wellen K.E., Hotamisligil G.S. (2003). Obesity-induced inflammatory changes in adipose tissue. J. Clin. Investig..

[28] Weyer C., Funahashi T., Tanaka S., Hotta K., Matsuzawa Y., Pratley R.E., Tataranni P.A. (2001). Hypoadiponectinemia in obesity and type 2 diabetes: Close association with insulin resistance and hyperinsulinemia. J. Clin. Endocrinol. Metab..

[29] Xu X.J., Gauthier M.S., Hess D.T., Apovian C.M., Cacicedo J.M., Gokce N., Farb M., Valentine R.J., Ruderman N.B. (2012). Insulin sensitive and resistant obesity in humans: AMPK activity, oxidative stress, and depot-specific changes in gene expression in adipose tissue. J. Lipid Res..

[30] Hardie D.G. (2008). AMPK: A key regulator of energy balance in the single cell and the whole organism. Int. J. Obes. (Lond.).

[31] Hwang J.T., Park I.J., Shin J.I., Lee Y.K., Lee S.K., Baik H.W., Ha J., Park O.J. (2005). Genistein, EGCG, and capsaicin inhibit adipocyte differentiation process via activating AMP-activated protein kinase. Biochem. Biophys. Res. Commun..

[32] Moon H.S., Chung C.S., Lee H.G., Kim T.G., Choi Y.J., Cho C.S. (2007). Inhibitory effect of (-)-epigallocatechin-3-gallate on lipid accumulation of 3T3-L1 cells. Obes. (Silver Spring).

[33] Hotamisligil G.S., Arner P., Caro J.F., Atkinson R.L., Spiegelman B.M. (1995). Increased adipose tissue expression of tumor necrosis factor-alpha in human obesity and insulin resistance. J. Clin. Investig..

[34] Hotamisligil G.S., Peraldi P., Budavari A., Ellis R., White M.F., Spiegelman B.M. (1996). IRS-1-mediated inhibition of insulin receptor tyrosine kinase activity in TNF-alpha- and obesity-induced insulin resistance. Science.

